# Exploring the perspectives of members of international tuberculosis control and research networks on the impact of COVID-19 on tuberculosis services: a cross sectional survey

**DOI:** 10.1186/s12913-021-06852-z

**Published:** 2021-08-12

**Authors:** Oluwatosin Nkereuwem, Esin Nkereuwem, Arnauld Fiogbe, Eno E. Usoroh, Abdou K. Sillah, Olumuyiwa Owolabi, Marc Tebruegge, Abdoulie Badjan, Beate Kampmann, Toyin Togun

**Affiliations:** 1 Medical Research Council Unit The Gambia at the London School of Hygiene and Tropical Medicine, Atlantic Road, Fajara, Banjul, The Gambia; 2 National Teaching Hospital for Tuberculosis and Respiratory Diseases, Cotonou, Republic of Benin; 3 Interactive Research and Development (IRD) - Nigeria, Folarin Coker House, Alausa, Ikeja, Lagos State Nigeria; 4 Department of Infection, Immunity and Inflammation, UCL Great Ormond Street Institute of Child Health, University College London, London, UK; 5 Department of Paediatrics, Royal Children’s Hospital Melbourne, University of Melbourne, Melbourne, Australia; 6 National Leprosy and Tuberculosis Control Programme (NLTCP), Ministry of Health, Banjul, The Gambia; 7 The Vaccine Centre, London School of Hygiene and Tropical Medicine (LSHTM), Keppel Street, London, UK; 8 The Tuberculosis Centre and the Faculty of Infectious and Tropical Diseases, London School of Hygiene and Tropical Medicine (LSHTM), Keppel Street, London, UK

**Keywords:** COVID-19, Pandemic, Tuberculosis, TB services, Survey

## Abstract

**Background:**

The COVID-19 pandemic has caused major disruption to healthcare services globally and has impacted on tuberculosis (TB) patients and TB diagnosis and treatment services both in low- and high-income countries. We therefore explored the perspectives of members of regional and international TB control and research networks to further understand TB service disruptions and compared the experiences of members from West African and European countries.

**Methods:**

This cross-sectional, explorative descriptive study was conducted from May to July 2020 using an open online survey with target respondents from both West African and European countries. The survey comprised discrete questions exploring challenges faced with TB screening, diagnosis, treatment, prevention, and changes implemented. Additionally, respondents were asked to provide recommendations for remedial actions.

**Results:**

We analysed responses from 124 respondents based in 29 countries located in Europe and West Africa. About half of the respondents reported challenges in delivering routine TB services during the COVID-19 pandemic, with over one third reporting having some form of guidance issued regarding maintaining delivery of routine TB services. Respondents emphasised the need for strengthening TB services especially in light of COVID-19 pandemic. Considerable similarities were found between the challenges experienced by TB professionals in both West African and European settings. Responses also highlighted the hidden challenges faced in some countries prior to the COVID-19 pandemic, especially in some West African settings where staff shortages and laboratory issues predated COVID-19.

**Conclusions:**

TB control and research professionals in West African and European settings experienced similar challenges to the delivery of TB diagnosis and treatment services due to the COVID-19 pandemic, and highlighted the need for clear communication of guidelines, prioritisation of routine TB service delivery, ongoing health education, and possible integration of TB and COVID-19 services to ensure that TB services are more resilient against the impact of the pandemic.

**Supplementary Information:**

The online version contains supplementary material available at 10.1186/s12913-021-06852-z.

## Background

The Coronavirus Disease-2019 (COVID-19) was declared a pandemic by the World Health Organization (WHO) on 11 March 2020 [1]. Since then, COVID-19 has caused extensive disruption to routine services for the control of both communicable and non-communicable diseases worldwide. This disruption, which could have far-reaching consequences, has continued well into 2021 and will most likely extend beyond [2, 3]. Low-income and high-income countries have been impacted by the pandemic, but the extent and pattern of the impact on routine health service delivery is still unfolding. Many low- and middle-income countries (LMIC) who were already battling high burdens of infectious diseases such as tuberculosis (TB) are likely to feel the impact of this disruption to a greater extent due to disruption of their health services [4, 5]. Similarly, many high-income countries (HIC) have experienced severely disrupted laboratory services due to the COVID-19 pandemic [6].

In recent years, some progress has been made in reducing the burden of TB, and ambitious targets have been set for reaching the WHO End TB Strategy targets of 90 % reduction in new TB cases and 95 % reduction in TB deaths by 2035, compared to 2015 [7]. However, many national TB control programmes are already facing major interruptions to their routine services, which could result in major setbacks, compounding the direct impact of COVID-19 [2, 4]. The impact of those disruptions has been estimated to have potentially resulted in up to 400,000 excess TB deaths in 2020, reverting the global situation to levels seen one decade ago [8]. Modelling studies also indicate that the COVID-19 pandemic could result in up to 6.3 million additional TB cases globally between 2020 and 2025 [3]. These consequences are envisaged to be global, with both LMIC and HIC having their share of the outcomes [6, 9].

Some of the causes for these outcomes include the additional pressure on health service resources due to the COVID-19 pandemic, reductions in the number of health facilities offering TB diagnostic and treatment services, reallocation of TB staff to the COVID-19 response, and concerns about stigma given the similarities in clinical presentation of TB and COVID-19 [3]. Taken together, the impact of COVID-19 on TB control services could slow or reverse progress towards WHO End TB Strategy targets, especially in high TB burden countries [10].

The WHO has issued several recommendations to member states to help mitigate the effects of the pandemic. These included maximising remote care and support for TB patients by using digital technologies, reducing the number of visits to health services during treatment, ensuring adequate infection prevention and control, and maintaining TB preventive treatment [3, 11].

With this survey, we sought to capture the experiences of healthcare professionals who work in TB-related services in West African and European countries, with a view to describe and compare the impact of the COVID-19 pandemic on routine TB services in both settings. We also sought to determine which measures and policies have been put into place to mitigate the effects of the pandemic on TB services, and to seek recommendations for reducing disruptions.

## Methods

### Study design, population and sampling method

We conducted a cross-sectional, exploratory descriptive online survey from May to July 2020 using a Google form designed to capture quantitative and qualitative data from respondents. The survey was carried out in accordance with research ethics and governance guideline of the UK National Health Services (NHS) Health Research Authority. Our target population were members of several international TB control and research networks. These included: (i) the West African Regional Network for TB Control (WARN-TB), which is a large network of national TB programme (NTP) managers from national Ministries of Health in West African countries; (ii) the TB network within the West African Networks of Excellence for TB, AIDS and Malaria (WANETAM) – a research capacity building network in West Africa that is funded by the European and Developing Countries Clinical Trials Partnership (EDCTP); (iii) the West African Paediatric TB Network (WApTBNet) – a subnetwork within WANETAM; and (iv) the Paediatric Tuberculosis Network European Trials Group (ptbnet) - a research network of > 300 clinicians and researchers based at 128 healthcare institutions across 31 European countries [12]. Members of these collaborative networks include policy makers, general physicians, paediatricians and paediatric subspecialists, nurses, clinical and laboratory research scientists with interest in adult and paediatric TB. We used a purposive sampling method with the aim to enrol as many participants as possible from each of the networks.

### Survey

We created an on-line survey that was aimed at addressing the impact of a novel global health challenge, and thus did not use any previously validated questionnaire. The online survey was designed in English by the study team comprising members with expertise in clinical and social science research related to TB treatment and control both in West Africa and Europe. The survey questions were initially piloted with five healthcare workers involved in TB control and research programme from The Gambia, Benin Republic, Nigeria and the United Kingdom, allowing us to refine the questions and options for responses. We then shared the final survey Google form to potential respondents through the broadcast email group addresses and WhatsApp chat groups of the respective networks.

The 11 survey questions aimed at understanding the experience and perspectives of the respondents regarding TB services in their respective localities and countries during the COVID-19 pandemic. The first ten questions captured the respondent’s country of work, profession, number of COVID-19 cases nationally at the time of completing the survey, followed by single-choice questions (“Yes”, “No” or “Don’t know”) with space to add open response text if “Yes” was selected. The last question was an open response question on what measures the respondent would like to be put in place for TB control during the COVID-19 pandemic in their respective country. The survey was administered in English. The Google survey form with the fields used for the subsequent extraction of quantitative and qualitative data is included in the Supplementary information (see Additional file 1).

### Ethical approval, data management and analysis

Research ethics committee review is not required for research involving healthcare staff recruited as research participants by virtue of their professional role (*Governance Arrangements for Research Ethics Committees, paragraph 2.3.14*) [13]. We therefore did not apply for a formal ethical approval. The survey had an introductory text stating its purpose and that the survey was completely anonymous, and informed consent was implied by respondents agreeing to participate in and completing the survey.

The quantitative and qualitative data obtained from the survey were downloaded into an Excel document. We analysed the quantitative data using Stata 16 (StataCorp, Texas, USA) for descriptive summary statistics (frequencies and percentages).

For the qualitative data, responses to the open-ended questions were analysed thematically using a deductive framework approach [14]. This involved reading and re-reading the dataset to develop familiarity with the data. We then iteratively developed a coding and thematic framework that identified important elements related to the survey questions. Initial themes were determined based on the prevalence of recurring responses across the dataset as well as the relevance of themes for answering the survey question. From this process, the overarching themes and corresponding sub-themes for each question were identified.

## Results

A total of 124 survey responses were received from respondents who reported about 29 countries. Given the networks approached, the survey respondents reported their experiences on the countries in in West Africa and in Europe as expected. A map showing the geographical distribution of survey respondents is included in the Supplementary information (see Additional file 2).

The majority of the respondents were from West Africa (*n* = 72; 58 %), and identified themselves as healthcare professionals, comprising doctors or nurses (*n* = 88; 71 %). The distribution of the survey respondents by occupation and country classification is shown in Table 1.

**Table 1:** Overview of the main occupation of survey respondents

Occupation	Overall (*n* = 124)	West Africa (*n* = 72)	Europe (*n* = 52)
Healthcare professional, n (%)	88 (71.0)	41 (56.9)	47 (90.4)
Academic researcher, n (%)	13 (10.5)	9 (12.5)	4 (7.7)
Public health official, n (%)	13 (10.5)	13 (18.1)	0
TB programme manager, n (%)	6 (4.8)	5 (6.9)	1 (1.9)
Lab-based scientist, n (%)	4 (3.2)	4 (5.6)	0

### Challenges within routine TB services

While half of the respondents reported that there were general challenges with routine TB screening and diagnosis during the COVID-19 pandemic, 56.5% of the respondents did not think it was more difficult to access routine TB treatment and prevention services . The distributions were similar in both West African and European settings (Table 2). However, a higher proportion of respondents from West Africa, relative to Europe, reported increased difficulty for presumptive TB cases to access TB diagnosis and care services (36.1% *vs.* 21.1%), and for patients on TB treatment to receive their TB drug supply including by direct observation of treatment (DOT) (25% *vs.*17.3%).

**Table 2:** Challenges in assessing routine TB screening, diagnosis, treatment and prevention services in countries during the COVID-19 pandemic

Variables	Overall (*n* = 124)	West Africa (*n* = 72)	Europe (*n* = 52)
Are there general challenges with routine TB screening and diagnosis?			
Yes, n (%)	62 (50.0)	37 (51.4)	25 (48.1)
No, n (%)	54 (43.5)	30 (41.7)	24 (46.1)
Don’t know, n (%)	8 (6.5)	5 (6.9)	3 (5.8)
Are there general challenges with routine TB treatment and prevention?			
Yes, n (%)	46 (37.1)	28 (38.9)	18 (34.6)
No, n (%)	70 (56.5)	39 (54.2)	31 (59.6)
Don’t know, n (%)	8 (6.4)	5 (6.9)	3 (5.8)
Is it more difficult for presumed TB patients to access diagnosis and care services?			
Yes, n (%)	37 (29.8)	26 (36.1)	11 (21.1)
No, n (%)	75 (60.5)	41 (56.9)	34 (65.4)
Don’t know, n (%)	12 (9.7)	5 (6.9)	7 (13.5)
Is it more difficult for TB patients to receive their TB drug supply or to have direct observation of their treatment?			
Yes, n (%)	27 (21.8)	18 (25.0)	9 (17.3)
No, n (%)	87 (70.2)	49 (68.1)	38 (73.1)
Don’t know, n (%)	10 (8.0)	5 (6.9)	5 (9.6)

Abbreviations: *TB* tuberculosis

**Fig. 1:**
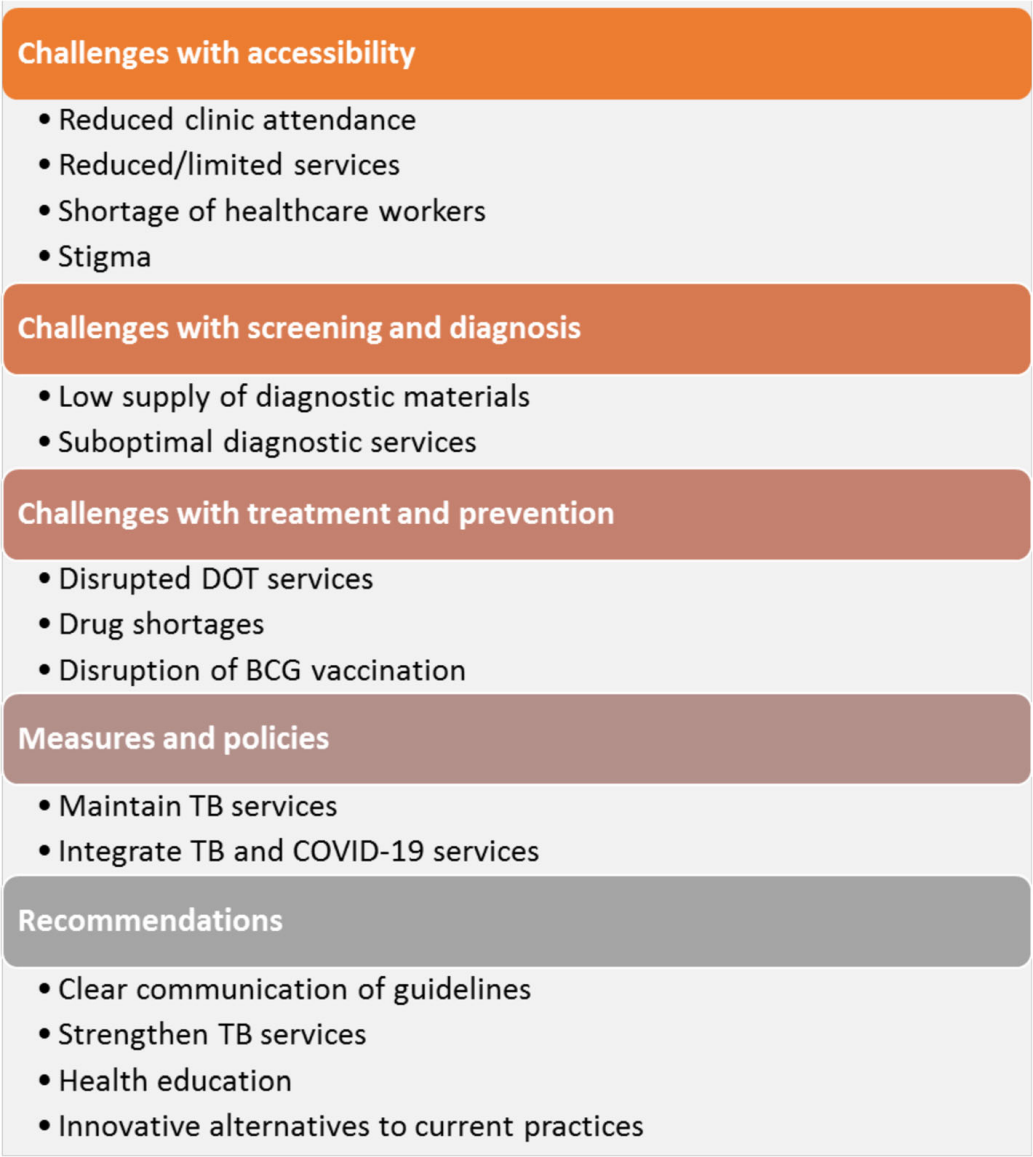
Key themes and subthemes that emerged from the open-ended questions

The narratives, however, that emerged from the open-ended questions suggested that there have been interruptions of routine TB services in both West African and European settings, as summarised in Figure 1, and the Supplementary information (see Additional file 2).

### Challenges with accessibility

Reduction in clinic attendance was reported from both settings, with respondents citing reasons such as *‘fear of COVID-19’*or restriction of movement as being responsible for this. In some instances where people with presumed TB sought care, they were unable to access care due to policies restricting visits to health facilities, or in other instances, complete closure of the health facility due to the pandemic. Concerning this issue, one respondent stated that: *“[this] has resulted in three deaths that I am aware of” (Healthcare Professional, UK).*

Furthermore, many respondents reported challenges with Healthcare Workers (HCWs) staffing in their settings. There were staff shortages in TB services because HCWs were unable to get to work due to the lockdown or due to being drafted into COVID-19-related work. Some HCWs were reluctant to work for fear of contracting COVID-19 due to lack of appropriate personal protective equipment (PPE).

Unique to the West African setting was the challenge of stigma, with HCWs said to be presumptively labelling any cough as possible COVID-19 and/or ‘*run away from anybody coughing’ *in some instances.

### Challenges with screening and diagnosis

Respondents from West Africa expressed challenges with laboratory services, mentioning issues such as lack of laboratory diagnostic materials, such as GeneXpert cartridges, and diversion of scarce TB laboratory equipment, such as GeneXpert machines, to COVID-19 services. While some participants reported shortages, others experienced laboratory equipment breakdowns. This typically resulted in a general slowing down of diagnostic services. As stated by one respondent, the reported challenges in some West African countries reflected problems that often existed prior to the emergence of COVID-19:

“[This] has been an issue even before the pandemic… However, the pandemic has worsened the situation…” (Public Health Official, Nigeria)

Conversely, the main challenges noted by European respondents were reductions in the frequency and quality of routine services, with TB screening services completely halted in some settings. Many respondents reported that they had to resort to the use of the interferon-gamma release assay (IGRA) for TB screening to avoid repeated face-to-face contact required for tuberculin skin testing.

### Challenges with treatment and prevention

Survey respondents from both West African and European countries reported a disruption in TB treatment monitoring by DOT, as well as shortages in both first line and second line TB medication. These shortages coupled with repurposing of some/several drug resistant-TB (DR-TB) treatment centres into facilities providing COVID-19 care resulted in DR-TB patients having serious challenges in accessing treatment in some locations.

“There is a shortage of few drugs (INH+Pyridoxine 100 mg combi tablet, rifampicin oral solution, etc).” (Academic Researcher, Germany).

“Shortages with some anti-TB drugs was experienced due to delay in delivery from central and zonal stores.” (Public Health Official, Nigeria).

In some European countries however, HCWs resorted to Video-Observed Therapy (VOT) and follow-up via telephone calls. In both settings, respondents stated that TB patients had to be given their medications for longer durations at a time, with limited follow-up visits to assess compliance.

Additionally, reports of disruption in BCG vaccination services were common in both West Africa and Europe, with up to 25% of eligible infants reported to have missed their neonatal doses of the vaccine in some settings. Some participants commented that contact tracing and migrant screening had slowed or even been suspended.

### Measures and policies to maintain routine TB services

Fewer than a third (33/124; 26.6%) of the respondents from both settings reported that specific guidance had been issued, at the national or regional level in their respective countries, regarding maintaining continuation of routine TB screening, diagnosis, prevention and treatment services during the pandemic. However, 29% of respondents in West Africa were unaware of any national or regional guidelines or policies, compared to less than 10% in Europe (Table 3).

**Table 3:** Country/region specific guidelines to maintain routine TB services

Variables	Overall (*n* = 124)	West Africa (*n* = 72)	Europe (*n* = 52)
Is there a country-specific guidance for routine screening, diagnosis, prevention or treatment of TB during the COVID-19 pandemic published?			
Yes, n (%)	33 (26.6)	21 (29.2)	12 (23.1)
No, n (%)	67 (54.0)	30 (41.6)	37 (71.1)
Don’t know, n (%)	24 (19.4)	21 (29.2)	3 (5.8)
Is there a region-specific guidance for routine screening, diagnosis, prevention or treatment of TB during the COVID-19 pandemic published?			
Yes, n (%)	26 (21.0)	17 (23.6)	9 (17.3)
No, n (%)	72 (58.0)	34 (47.2)	38 (73.1)
Don’t know, n (%)	26 (21.0)	21 (29.2)	5 (9.6)

Abbreviation: *TB* tuberculosis

Some of the measures and policies put in place to ensure continuation of services included Standard Operating Procedures (SOP) and guidelines at national and regional levels. The respondents also highlighted that there were policies to guide the integration of services for TB and COVID-19, with awareness campaigns on infection prevention and control, appropriate use of PPE, and safety in the work environment targeted at health care workers. In both settings, respondents commented that patients were given their TB medication supplies for longer periods than usual to reduce the number of visits to health facilities. In most instances, these policies were disseminated by mail, email or social media, and in a few instances the respondents provided links to relevant websites [15, 16].

### Recommendations made by study respondents

There were fewer responses from Europe (37%) compared to West Africa (56%) on the question of recommendations for workable solutions and lessons learnt during the pandemic. Qualitative responses from West African countries underscored the need for clear communication of recommendations and guidelines at national and regional levels to guide the TB delivery services, especially during potentially disruptive situations such as the COVID-19 pandemic. Conversely, no respondent from Europe mentioned this recommendation.

*“I would like that there was a statement/guidance from the NLTP to guide the operation/sustenance of TB services during this period.” (Healthcare Professional, The Gambia)*

*“I think there should be a clear guidance for routine screening, diagnosis and treatment for tuberculosis in this COVID-19 pandemic to all facilities.” (Laboratory-based Researcher, Ghana)*

Another recommendation highlighted by respondents from both settings was the need for strengthening TB services by ensuring availability of diagnostic tools and medication, as well as supporting staff wellbeing, notwithstanding the pandemic. Many specifically mentioned the necessity for this to continue despite the COVID-19 pandemic. Also highlighted was that TB programmes needed to ensure that staff within TB services are viewed as “essential”, ‘*so as to protect them from redeployment to emergency services as part of the COVID-19 response*’* (Healthcare Professional, UK).*

*“The urgency of uncompromised TB diagnostics/treatment and follow up should be a national concern… Diagnostics and interprofessional exchange should not be compromised for TB patients during the COVID pandemic.” (Healthcare Professional, Austria)*

*“[There should be] encouragement of TB programme officers and other health care professionals by providing enough PPE and other incentives for them to be able to do more.” (Healthcare Professional, Nigeria)*

As many as 25% of respondents from West African countries recommended that TB programmes should take advantage of the current situation for active case finding by testing or screening all COVID-19 suspects for TB. Some of the suggestions offered included ‘*applying the cough questionnaire to screen for TB’* and ‘*concurrent TB screening among suspected COVID-19 patients who present with cough*’.

There was also emphasis on the need for intensified health education to remind the world ‘*not to forget TB’* and to make people aware that ‘*chronic cough may not just be COVID’*. As stated by one respondent:

*“There needs to be more public education on TB since COVID-19 has overshadowed the education on other health conditions” (Healthcare Professional, Ghana).*

It was also proposed by several respondents in both settings that there is need to consider alternatives to the current face-to-face consultations and DOT practices in the post-COVID era. Their suggestions included virtual medical consultations, remote monitoring of treatment, and home visits for delivery of medication, even beyond the pandemic. As stated by one respondent, we should *‘remember the value of remote monitoring for post-COVID to avoid unnecessarily long in-patient stay for observation.’ (Healthcare Professional, UK)*

## Discussion

Our survey of 124 members of international TB control and research networks provides evidence of the impact of COVID-19 on routine TB services in low-income as well as in high-income settings from those at the front line of TB services. We found considerable similarities in the challenges to delivery of routine TB services experienced by service providers in both settings. Three main challenges were identified, namely, challenges with accessibility, challenges with screening and diagnosis, and challenges with treatment and prevention. The survey highlights some specific measures and policy adaptations that were implemented to reduce disruptions. We also detail the recommendations offered by members of the networks, many of whom are frontline healthcare workers in TB diagnosis and treatment services, to mitigate the impact of the pandemic on TB services. These include the need for clear communication on maintenance of routine services during similar situations, the need to re-prioritise routine TB services to ensure that they continue without interruption despite the COVID-19 pandemic, and the need for continued health education of the public, and the need for innovative alternatives to the current TB care practices.

Our findings are similar to that of a recent report on a survey by Khan *et al*., which also found that access to TB services for both healthcare providers and patients had been substantially affected during the pandemic [2]. Although that survey was exclusively conducted in LMICs, findings from a broader range of HCWs in our study suggest that both low- and high-income settings had very similar experiences of reduced access to TB services, with consequent TB-related deaths reported in some instances. These findings suggest that it is imperative to re-focus resources to TB services to mitigate the impact of COVID-19 on TB, where these services are based [8].

Our survey findings also suggest that due to the pandemic, routine TB preventive services such as screening for latent TB infection and administration of BCG vaccine were adversely affected. These changes were as forecast by researchers who anticipated that TB services would become more focused on the care of active TB disease rather than prevention [9]. Deployment to COVID-19 services, HCWs being off duty due to sickness or self-isolation, and the concern that social contact within clinical spaces may result in more COVID-19 transmission are reasons for these observations [9]. We found this to be the case in our survey, in both West Africa and Europe.

The pandemic has also exposed some of the pre-COVID-19 frailties in the health systems. For example, the pre-existing regular anti-TB therapy stock-outs in West Africa and some European countries became exacerbated and more apparent due to the disruption of supply chains worldwide [17, 18]. Similarly, in many West African countries where there have been pre-existing weak healthcare systems, the diversion of political will and scarce human resources to the COVID-19 response have further hampered routine TB services [3].

Fewer than a third of the respondents reported that they were aware of guidance or policy adaptations to maintain TB services during the pandemic. This finding suggests a need for facilitated information sharing from central, national or regional offices to frontline HCWs, as recommended by our respondents [2]. Adaptations, particularly in West Africa, could include incorporating TB and COVID-19 services, and concerted dissemination of COVID-19 and TB health education and stigma-reduction messages [19]. Integrating knowledge and awareness of TB into the health education for frontline HCWs and community education on behavioural practices such as cough etiquette and infection prevention and control may ultimately contribute to the reduction of TB transmission [20]. These, in addition to the need to ensure that TB services remain resilient throughout pandemic situations are crucial if the End TB targets of 2030 are to be met [21, 22].

As recommended by the respondents, there is a need for innovative alternatives to the traditional DOTs and frequent clinic visits for TB care which were being practiced in the pre-COVID-19 era. Notably, the COVID-19 situation presents an opportunity for a re-evaluation of the current policies and practices of delivery of TB care. The need to reduce clinic attendance during the pandemic may equally present the opportunity to strengthen home-and-community-based care and exploring virtual means of medical consultation and care [23].

Our survey highlights the nature of the impact of COVID-19 on TB service delivery with European and West African countries similarly affected. As experiences are shared between countries and across income settings, solutions and policies applied in one setting could be adapted for use in another [24, 25]. Additionally, the COVID-19 pandemic remains fluid, and ongoing changes imply that the recommendations might require many adaptations.

Our survey has some notable limitations: the overall small sample size may not be fully representative of the populations surveyed. The total number of respondents who filled out the survey, and even smaller number who responded to the qualitative aspects might limit the generalisability of our findings. Considering that the survey was conducted during the peak of the first wave of the pandemic, we acknowledge that if repeated now, the findings might be different. Furthermore, in the context of the ongoing COVID-19 pandemic, to encourage participation and to avoid diverting substantial time away from clinical duties, we decided to focus on only a relatively small number of key variables. Also, we provided examples of answers to some of the qualitative aspects of the survey as guides for our respondents. Taken together, these may have limited the spread and diversity of responses obtained in the open-ended questions. We also recognise that the availability of the survey in English language only may have led to the self-selection of participants, and we may have missed professionals who do not speak English.

## Conclusions

Our survey of frontline professionals highlights the similarities in challenges in accessing and maintaining routine TB services during the COVID-19 pandemic in West African and European settings. Their recommendations to mitigate the impact include prioritisation of routine TB service delivery to ensure that they are more resilient against future epidemics, ongoing health education, and integration of healthcare delivery services. They also stressed the need for clear communication of guidelines in the future and suggested the need for innovative ways of delivering TB care. These concerted efforts are needed to ensure that the targets of the WHO’s End TB Strategy can be achieved.

## Supplementary Information

**Additional file 1.** Google survey form with the fields used for the subsequent extraction of quantitative and qualitative data.

**Additional file 2. **Map showing the geographical distribution of survey respondents.Map was created with MapChart.net; the created map is licensed under a Creative Commons Attribution-ShareAlike 4.0 International License: https://creativecommons.org/licenses/by-sa/4.0/

**Additional file 3. **Thematic analysis of the responses to open-ended questions by survey respondents according to country category.

## Data Availability

The datasets generated and/or analysed during the current study are available from the corresponding author on reasonable request.

## Abbreviations

BCG: Bacille Calmette-Guérin
COVID-19: Coronavirus Disease-2019
DOT: Directly-Observed therapy
DRTB: Drug-Resistant Tuberculosis
EDCTP: European and Developing Countries Clinical Trials Partnership
HIC: High-Income countries
IGRA: Interferon Gamma Release Assay
LMIC: Low- and Middle-Income Countries
NHS: National Health Services
NTP: National Tuberculosis Programme
PPE: Personal Protective Equipment
ptbnet: Paediatric Tuberculosis Network European Trials Group
SOP: Standard Operation Procedures
TB: Tuberculosis
VOT: Video-Observed Therapy
WANETAM: West African Network for TB, AIDS and Malaria
WApTBNet: West African Paediatric TB Network
WARN-TB: West African Regional Network for TB Control
WHO: World Health Organization
