# Supplementary material for: Exploring the perspectives of members of international tuberculosis control and research networks on the impact of COVID-19 on tuberculosis services: a cross sectional survey
Source: BMC Health Serv Res. 2021 Aug 12;21:798. doi: 10.1186/s12913-021-06852-z (PMC8358254; doi:10.1186/s12913-021-06852-z)
Supplement: Supplementary file 1 — Additional file 1. Google survey form with the fields used for the subsequent extraction of quantitative and qualitative data. [file 12913_2021_6852_MOESM1_ESM.pdf]

TB control in the era of COVID-19

Please, let us know your experience and thoughts on TB control in the era of the COVID-19 pandemic in your country. This form is COMPLETELY ANONYMOUS. We want to use the data collected through this form to investigate and describe TB control in the era of COVID-19 pandemic, from the perspectives of healthcare professionals, TB programme managers and researchers who are members of key regional and international TB control and research networks in Europe and West Africa. The data will not be shared with any third parties or used for any other purposes. Thank you for your cooperation.

*Required

1. 1. Which country are you reporting from? *

____

2. 2. What is the most fitting description of your job? *

Mark only one oval.

- ◯ HEALTHCARE PROFESSIONAL (i.e. DOCTOR, NURSE; Hospital or Community based
- ◯ PUBLIC HEALTH OFFICIAL
- ◯ TB PROGRAMME MANAGER
- ◯ ACADEMIC RESEARCHER (University or Research Institute based)
- ◯ LABORATORY BASED RESEARCHER
- ◯ PRIVATE CARE PROVIDER
- ◯ Other: _______________________________

3. If you clicked 'Other', please specify

____

4. 3. When was the first case of COVID-19 reported in your country? (estimated date is sufficient) *

____

Example: 7 January 2019

5. 4. How many COVID-19 cases have been recorded in your country by the time you fill this in? *

____

6. 5. Are you aware of any GENERAL problem with routine SCREENING & DIAGNOSIS of TB in your country during the COVID-19 pandemic? (e.g. administration problems; staff shortages; challenges with diagnosis including availability of Xpert; plans to use Xpert for COVID-19 diagnosis; social/logistical barriers; Etc.): *

Mark only one oval.

- YES
- NO
- DON'T KNOW

7. If 'Yes', please provide some detail below:

____
____
____
____
____

8. 6. Are you aware of any GENERAL problem with routine TREATMENT & PREVENTION of TB in your country during the COVID-19 pandemic? (e.g. drug shortages; problems with direct observation of TB treatment; staff shortages; challenges with BCG vaccination; challenges with provision of TB preventive therapy; Etc.): *

Mark only one oval.

- YES
- NO
- DON'T KNOW

9. If 'YES', please provide some detail below:

____

____

____

____

____

10. 7. SPECIFICALLY, are you aware if presumed TB patients (i.e. patients with chronic cough) have any difficulty in accessing TB diagnosis and care services in your country during the COVID-19 pandemic? *

Mark only one oval.

- YES
- NO
- DON'T KNOW

11. If 'Yes', please provide some detail below:

____

____

____

____

____

12. 8. SPECIFICALLY, are you aware if TB patients on anti-TB treatment have any difficulty in receiving their TB drugs supply or in direct observation of their treatment in your country during the COVID-19 pandemic? *

Mark only one oval.

- YES
- NO
- DON'T KNOW

13. If 'Yes', please provide some detail below:

14. 9. Has any guidance for routine SCREENING, DIAGNOSIS, PREVENTION or TREATMENT of Tuberculosis during the COVID-19 pandemic been issued at the NATIONAL level in your country? * *

Mark only one oval.

- YES
- NO
- DON'T KNOW

15. 10. Has any guidance for routine SCREENING, DIAGNOSIS, PREVENTION or TREATMENT of Tuberculosis during the COVID-19 pandemic been issued at the REGIONAL and/or LOCAL level in your country? * *

Mark only one oval.

- YES
- NO
- DON'T KNOW

16. If 'YES' to questions 8 and/or 9 above, please share details below of any such NATIONAL, REGIONAL or LOCAL guideline and/or special measures that have been put in place during the COVID-19 pandemic in your country (including any useful links):

17. 11. Are there any other special measures that 'YOU' WOULD LIKE TO BE PUT IN PLACE for TB control during the COVID-19 pandemic in your country?

This content is neither created nor endorsed by Google.

Google Forms
