# Supplementary material for: Exploring the perspectives of members of international tuberculosis control and research networks on the impact of COVID-19 on tuberculosis services: a cross sectional survey
Source: BMC Health Serv Res. 2021 Aug 12;21:798. doi: 10.1186/s12913-021-06852-z (PMC8358254; doi:10.1186/s12913-021-06852-z)
Supplement: Supplementary file 2 — Additional file 2. Map showing the geographical distribution of survey respondents.Map was created with MapChart.net; the created map is licensed under a Creative Commons Attribution-ShareAlike 4.0 International License: https://creativecommons.org/licenses/by-sa/4.0/ [file 12913_2021_6852_MOESM2_ESM.pdf]

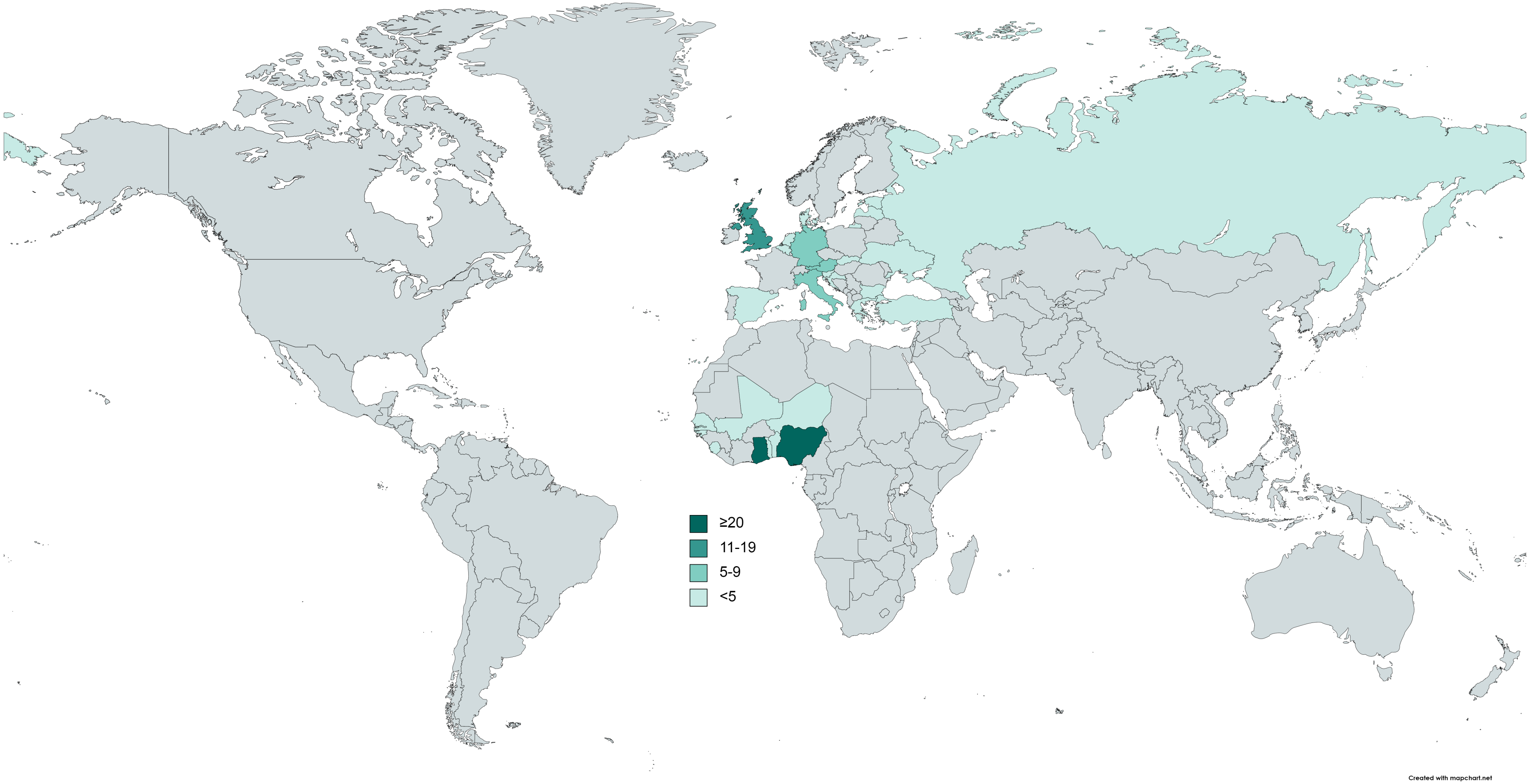

Supplementary Figure 1: Map showing the geographical distribution of survey respondents. Map was created with MapChart.net; the created map is licensed under a Creative Commons Attribution-ShareAlike 4.0 International License: https://creativecommons.org/licenses/by-sa/4.0/
