# Supplementary material for: Exploring the perspectives of members of international tuberculosis control and research networks on the impact of COVID-19 on tuberculosis services: a cross sectional survey
Source: BMC Health Serv Res. 2021 Aug 12;21:798. doi: 10.1186/s12913-021-06852-z (PMC8358254; doi:10.1186/s12913-021-06852-z)
Supplement: Supplementary file 3 — Additional file 3. Thematic analysis of the responses to open-ended questions by survey respondents according to country category. [file 12913_2021_6852_MOESM3_ESM.docx]

**Additional file 3:** Thematic analysis of the responses to open-ended questions by survey respondents according to country category

| **Theme** | ***Quotes from HIC*** | ***Quotes from LIC*** |
| --- | --- | --- |
| **Challenges with accessibility** | | |
| Reduced clinic attendance | ‘During the initial lockdown patients did not dare to come to the hospital, this has "normalized" now’ *(Healthcare Professional, Germany).*  ‘Patients/contacts worried of attending due to COVID-19, despite reassurances that we are open’ *(Healthcare Professional, UK).*  ‘People do not go to the doctor because of COVID-19 fear.’ *(Academic Researcher, Greece).*  ‘There are concerns that many patients with symptoms of non-COVID disease, such as TB, are not seeking help either because they are following advice to self-isolate or because of fear of coming to hospital. I have experience of this in children with TB symptoms whose families will not bring to hospital for assessment’ *(Health Professional, UK).*  ‘Potentially 111 will tell people with cough to stay at home’ *(Health Professional, UK).* | ‘…the number of clients visiting hospitals for their pre-scheduled appointments has declined significantly leading to reduction in TB screening … due to the lockdown situation’ *(Public Health Official, Nigeria).*  ‘Due to partial lockdown instituted, out-patients department turnouts were low decreasing the number that have presumed TB’ *(Healthcare Professional, Ghana).*  ‘The movement restriction due to COVID-19 pandemic, plus the policy of most health facilities and hospitals to only take in emergency cases has drastically reduced hospital attendance by about 80%’ *(Public Health Official, Nigeria).*  ‘OPD screening has stopped’ *(Public Health Official, Ghana)*.  ‘Patients on TB medications are not coming for them due to the pandemic condition’ *(Healthcare Professional, Ghana).*  ‘Restriction of movement across the country has significantly reduced patient volume at healthcare centres’ *(TB Programme Manager, Nigeria).* |
| Reduced/limited services | ‘We can provide only urgent visit, so migrant screenings are stopped’ *(Healthcare Professional, Italy).*  ‘Only emergency or for people on treatment out-patients visits were permitted’ *(Healthcare Professional, Estonia).*  ‘Patients did not get an appointment easily, some medical practices were closed since no protective clothing was available’ *(Healthcare Professional, Germany).* | ‘Some DOTs centres are closed making access to investigations difficult’ *(Healthcare Professional, Nigeria).* |
| Healthcare workers | ‘Staff shortage due to obligations with COVID-19 surveillance as well as quarantine of affected health workers’ *(Healthcare Professional, Germany).*  ‘Less staff as TB nurses [have been] recruited to respiratory wards…’ *(Healthcare Professional, UK).* | ‘Because of lockdown health workers find it difficult to get to work’ *(Public Health Official, Nigeria)*.  ‘HCWs are afraid to take samples for TB if Covid-19 has not been ruled out. HCWs working with TB patients have been recruited to the Covid-19 teams’ *(Healthcare Professional, Ghana).*  ‘Laboratory staff were reluctant to do TB testing because of unavailability of PPEs’ *(Healthcare Professional, Ghana).*  ‘Because many health workers have gotten infected with COVID-19, other health workers are reluctant to attend to patients who present with respiratory symptoms for the fear of getting infected’ *(TB Programme Manager, Nigeria).*  ‘The same team take care for both [COVID-19 and TB] cases with some few extra hand from the various wards and units’ *(Public Health Official, Ghana).* |
| Stigma | - | ‘There is a lot of fear of contagion amongst health care workers as quite a number have died after contracting Covid-19. Due to the cross-cutting symptoms of TB and Covid-19, there has been stigmatization of presumptive TB patients in facilities not providing covid-19 services’ *(TB Programme Manager, Nigeria).*  ‘Health staff run away from anybody coughing’ *(Public Health Official, Nigeria).*  ‘Any individual with cough is presumed to have COVID and stigmatized by most [people]’ *(Public Health Official, Nigeria).* |
| **Challenges with screening and diagnosis** | | |
| Low supply of diagnostic materials | - | ‘Currently there is shortage of Xpert cartridges for TB in my region. The government is planning to buy some cartridges for about 100 GeneXpert testing sites to compliment the research labs in the country’ *(Healthcare professional, Ghana).*  ‘…non-working Gene-Xpert machines’ *(Healthcare professional, Nigeria).*  ‘There is an ongoing plan by ministry of health to use Xpert machines for COVID-19 diagnosis.’ *(Lab-based scientist, Nigeria).* |
| Suboptimal diagnostic services | ‘Contact tracing of diagnosed cases may not be conducted as it should. Screening of high-risk children and adults may lag behind’ *(Academic Researcher, Greece).*  ‘… going back to IGRA not Mantoux to avoid face-to-face appointments’ *(Healthcare Professional, UK).*  ‘we have not carried out TB screening in last 8 weeks’ *(Healthcare Professional, UK).* | ‘Production of sputum samples has become difficult especially in children where sputum induction is no longer done. Danger of aerosol generation. Detection rates have gone down’ *(Healthcare Professional, Ghana).*  ‘Sometimes GeneXpert result takes a long time sometimes more than a week to be ready’ *(Healthcare Professional, Ghana).* |
| **Challenges with treatment and prevention** | | |
| Disrupted DOT services | ‘Medication supplies are given for longer periods of times, there is a relaxation on follow up and monitoring of compliance’ *(Healthcare Professional, UK).*  ‘DOT has been interrupted; some replacement with VOT’  *(Healthcare Professional, UK).* | ‘Direct observation of clients has been a challenge for staff’ *Academic Researcher, Ghana).*  ‘[We] don’t do DOT anymore, [we] give all drugs to patients’ *Public Health Official, Sierra Leone).*  ‘Because of [the lockdown], TB patients on treatment cannot come in for their DOTS or drug pick-up. In addition, most states have redirected the use of their DRTB Treatment centres to covid-19 isolation centres (because of existing IPC measures) thus presenting a challenge to initiating DRTB treatment for those co-morbid or very ill patients who need hospitalisation to commence DRTB treatment’ *(TB Programme Manager, Nigeria).* |
| Drug shortages | ‘There is a shortage of few drugs (INH+Pyridoxine 100 mg combi tablet, rifampicin oral solution, etc)’ *(Academic Researcher, Germany).*  ‘Drug shortages’ *(Healthcare Professional, Austria).* | ‘We have stock outs of DRTB medicine especially linezolid and it is being substituted with pyrazinamide though not officially communicated’ *(Healthcare Professional, Nigeria).*  ‘Shortages with some anti TB drugs was experience due to delay in delivery from central and zonal stores. Direct observation of treatment was not possible in a lockdown areas*’ (Public Health Official, Nigeria).* |
| Disruption of BCG vaccination | ‘BCG catch up clinic not working so 25% of eligible Neonates not getting BCG’ *(Healthcare Professional, UK).*  ‘BCG vaccination paused in some areas’ *(Healthcare Professional, UK).*  ‘Since selective vaccination of newborns … takes place … in an aggregated manner in order to optimize BCG vaccine utilization, the lockdown measures posed some organization issues’ *(Healthcare Professional, Slovenia).* | ‘Parents are not sending children for vaccinations especially if BCG is not given at birth.’ *(Healthcare Professional, Ghana).*  ‘[There is] reduction in BCG [vaccination] at birth’ *(Public Health Official, Sierra Leone).* |
| **Measures and policies** | | |
| Maintain TB services | ‘[There was an] NHS letter to all services, to keep TB services running’ *(Healthcare Professional, UK).* | ‘The NTBLCP has issued specific guidelines for the programme activities in the phase of COVID-19 pandemic’ (*TB Programme Manager, Nigeria).*  ‘Letter from the Ministry of Public Health notifying an endowment of 1 month of treatment for tuberculosis patients’ (*TB Programme Manager, Niger).* |
| Integrate TB and COVID-19 services | - | ‘The main guidance is that of integration of services for TB and covid-19. A lot of awareness is also being created for health care workers on IPC, appropriate use of PPEs and staying safe in the work environment’ (*TB Programme Manager, Nigeria).* |
| **Recommendations** | | |
| Clear communication of guidelines in the future |  | “I would like that there was a statement/guidance from the NLTP to guide the operation/sustenance of TB services during this period.” *(Healthcare Professional, The Gambia)*  “I think there should be a clear guidance for routine screening, diagnosis and treatment for tuberculosis in this COVID-19 pandemic to all facilities.” *(Laboratory-based Researcher, Ghana)* |
| Strengthen TB services | ‘The urgency of uncompromised TB diagnostics/treatment and follow up should be a national concern, diagnostics and interprofessional exchange should not be compromised for TB patients during the COVID pandemic, enough tb experts should be present or consultable in TB-outpatient clinics in case of staff shortages and quarantine’ *(Healthcare professional, Austria).*  ‘Protection of TB teams to ensure vital staff are not redeployed without adequate alternative arrangements in place’ *(Healthcare Professional UK).*  ‘Ensuring the availability of reserve anti-tuberculosis drugs’ *(Healthcare Professional, Slovakia).* | ‘Since now there is intensive screening for COVID at entry to all facilities, the public health team should take advantage and be present to apply the cough questionnaire to screen for TB as well’ *(Healthcare Professional, Ghana).*  ‘COVID-19 should be used as a form of active case finding by testing and or screening all COVID suspects for TB’ *(Public Health Official, The Gambia).* |
| Health education | ‘Advice NOT to forget TB, make people aware chronic cough may NOT just be COVID, lots of other diagnoses to investigate’ *(Healthcare Professional UK).* | ‘There needs to be more public education on TB since COVID-19 has overshadowed the education on other health conditions’ *(Healthcare Professional, Ghana).*  Health promoters must continue to educate people both conditions to create awareness’ *(Public Health Official, Ghana).* |
| Innovative alternatives to current practices | ‘Remember value of remote monitoring for post-COVID: avoidance of unnecessarily long inpatient stay for 'observation’’ *(Healthcare Professional UK).* | ‘Online platforms for patients to assess care from diagnosis, treatment and other services’ *(Healthcare Professional, Nigeria).*  institute home delivery of medicines and reorganisation of care services’ *(Public Health Official, Ghana).*  Widespread provision of structure for virtual medical consultation’ *(Healthcare Professional, Nigeria).* |
